# Poor-Law Infirmaries and the War

**Published:** 1914-08-15

**Authors:** 


### Poor-Law Infirmaries and the War.

*To the Editor of* The Hospital.

Sir,—In all probability a great number of beds will be demanded for the treatment of injured and those invalided in the present war. I suggest that Poor-Law authorities might offer beds in some of the up-to-date infirmaries of the Metropolis, transferring chronic cases to other institutions where they would still receive attention suitable for their needs.

Of course, no question of "pauper" treatment should be associated with the use of the Poor-Law hospitals, which, I think, are eminently more suited than schools and such buildings.—Yours faithfully,

Infirmary Officer.

[It is interesting to note that this letter was received at our office after the leading article on the same subject was in type. This indicates the feeling inspiring Poor-Law infirmary medical superintendents and their staffs. If acted upon, might it not give, apart from immediate help to the nation, the *coup de grace* to the already practically nominal "pauper qualification"?—Ed. The Hospital.]

